# Alcohol free? An analysis of UK and Scottish Government obesity policies’ engagement with alcohol 1999–2023

**DOI:** 10.1186/s12889-024-19852-5

**Published:** 2024-09-05

**Authors:** Callum Young, Benjamin Hawkins

**Affiliations:** MRC Epidemiology Unit, Box 285 Institute of Metabolic Science, Cambridge Biomedical Campus, University of Cambridge School of Clinical Medicine, Cambridge, CB2 0QQ UK

**Keywords:** Obesity policy, Alcohol policy, Public health, Alcohol industry, Scotland, UK

## Abstract

**Background and aims:**

Alcoholic beverages can be highly calorific yet remain largely absent from obesity policy debates. This article seeks to identify how Scottish and English obesity policies have engaged with the issue of alcohol consumption since devolution.

**Methods:**

Obesity policy documents for England and Scotland from 1999 to 2023 were thematically analysed to identify their engagement with alcohol consumption. A stakeholder analysis was undertaken to identify key public health actors and commercial sector policy actors in the debate regarding the inclusion of alcohol in obesity policy. Their engagement with the issue of alcohol as an obesity policy issue was assessed through thematic analysis of consultation responses, along with documents, press releases, reports and other statements on policy (e.g. blog posts) available on stakeholder websites.

**Results:**

While alcohol was recognised as a risk factor for obesity within obesity policy documents, no specific measures to address this issue were identified until a consultation on mandatory calorie labelling on alcoholic beverages was proposed in 2020. Engagement with alcohol in the policy documents was mainly limited to voluntary and self-regulatory measures favoured by industry actors who portrayed themselves as a key part of the policy solution. They used the policy focus on childhood obesity as a pretext to exclude alcoholic drink from fiscal and labelling measures. Public health NGOs, by contrast, argued that obesity measures such as mandatory calorie labelling and other obesity policies should be extended to alcoholic beverages.

**Conclusion:**

There is an insufficient engagement with alcohol as an obesity policy issue within policy documents and an over-reliance on voluntary and industry-partnership approaches. Alcoholic beverages and reduced alcohol products are excluded from beverage taxes and labelling requirements in ways which are hard to justify. As with other areas of public health policy, this represents an industry-favoured policy agenda, opposed by health NGOs. Further research is needed to understand the influence of these actors on the engagement of obesity policy with alcohol.

## Introduction

Obesity is associated with a range of health conditions including an increased risk of hypertension, type 2 diabetes and various cancers, and is estimated to cost the United Kingdom’s National Health Service (NHS) between £4.2 billion to £6.1 billion a year [1]. In 2021 43% men and 32% women in England had a body mass index (BMI) of 30 or higher and were thus classified as obese [2]. A similar pattern is evident in Scotland where the adult obesity prevalence was 30% in 2021 and 24.1% of primary 1 children were at risk of being overweight and obese [3]. Obesity was first formally recognised by the UK government as a policy problem in 1991 [4]. The intervening three decades have seen the publication of 19 obesity strategies and policy documents by the UK – and, since 1999, the Scottish – governments reflecting both the worsening nature of the obesity crisis and increasing calls for effective policy responses [4].

The key contributor to rising obesity levels are unhealthy diets comprising of energy-dense foods and beverages, high in fat and/or sugars [5]. Alcoholic drinks often have high calorie and sugar contents, and their consumption has been identified as a contributory factor for weight gain and associated chronic diseases [6–10]. Alcoholic drinks can also lead to increase food intake through the stimulation of appetite and lowering inhibitions and can negatively impact the quality and quantity of sleep which may also increase the risk of obesity [11]. Consequently, there is significant potential to decrease population-level calorie intake and obesity via alcohol-focussed policy measures [6, 7, 12]. Such an approach would be in keeping with previous attempts to reduce consumption of “dead calories” consumed via alcohol-free, sugar-sweetened beverages. However, comparatively little attention has been paid to alcohol within obesity policy. While Scotland’s most recent obesity strategy [13] focussed on restricting promotion of food and drink high in fat, sugar or salt (HFSS), restrictions on advertising, voluntary product labelling and product reformulation, alcohol products are not included in any of these measures. Similarly, England’s latest obesity strategy [14] did propose a consultation on alcohol calorie labelling. However, at the time of writing, it has yet to be released, suggesting it remains a low priority for the UK government.

It is estimated that excessive alcohol consumption contributes to up to 3 million avoidable deaths per year [15], and is associated with substantial health inequalities in the UK, with the most marginalised socioeconomic groups experiencing 1.5-2 fold higher alcohol-related mortality [16]. Excessive alcohol consumption is especially problematic in Scotland where a quarter of the population exceed the recommended guidelines of 14 units per week [17] and, in 2021, alcohol was an underlying cause of 1,245 deaths and 35,187 hospital admissions [18]. Consequently, both the UK and Scottish Governments have recognised alcohol as an important public policy issue, introducing specific policies and strategies designed to tackle the health and wider social consequences of alcohol consumption [19]. However, these generally do not engage specifically with the role of alcohol as a risk factor for obesity. This perhaps reflects the range of other risks associated with drinking (e.g. drink driving), and a (perhaps tacit) assumption that issues relating to the caloric content of alcoholic drinks would be more logically addressed in the context of obesity policy. Yet, as noted above, alcohol features in only limited ways in obesity strategies in both Scotland and England.

Previous studies have found evidence of both significant industry influence over public health policy agendas and outputs [20], but also an ability of public health advocates to overcome industry resistance to achieve policy change (e.g. on minimum unit pricing of alcohol) [21]. Consequently, the ways in which policy actors have engaged with these issues is a potentially key factor in explaining the (dis)engagement of UK and Scottish obesity policies with alcohol consumption.

Given the importance of alcohol as an obesity risk factor [22] and the high levels of alcohol consumption within the UK, the lack of focus on alcohol-related measures from obesity policies in both Scotland and England appears anomalous and in need of further enquiry. This article has two objectives. First, it seeks to document if and how obesity policies in Scotland and England have engaged with the issue of alcohol consumption since the devolution of health policy in 1999. This placed responsibility for health policy with the Scottish Government thus creating the possibility for policy divergence with England [23].^1^ Second, recognising the important role of both public health and commercial actors in setting policy agendas [24], it examines the advocacy positions of key obesity policy stakeholders, and the extent to which they engaged with the issue of alcohol, through a thematic analysis of their engagement in obesity policy debates.

## Methods

This article presents the results of a thematic analysis of UK alcohol policy documents and interventions in obesity policy debates by key public health actors and commercial stakeholders from 1999 to 2023. Theis and White’s [4] catalogue of obesity policies in England was used to identify relevant policy documents and consultations in England, supplemented by additional searches on the “gov.uk” website. Documents for Scotland were found by searching the “gov.scot” website, inputting “Obesity” into the search bar, selecting the “Topic” as “Health and Social Care” and then selecting “Policy papers” and “Consultations” for the “Document type”. One English and 4 Scottish obesity policy documents solely focused on increasing the population’s physical activity levels to reduce obesity and were excluded from the data set as they did not assess diet or food and drink consumption as a risk factor of obesity. In addition to obesity specific documents, public health strategies with a specific focus on obesity were included in the study. This resulted in 14 UK Government obesity policy documents and 5 Scottish Government policy documents included in the dataset (see Table 1).

**Table 1:** UK & Scottish Government obesity-related policy documents from 1999 to 2023

Policy (Government)	Year	Type	Description	References to alcohol as an obesity policy issues
Reducing Health Inequalities: An Action Report (UK)	1999	Public health strategy including obesity	The key aim of this health strategy was to improve the health of the worst off in society. The strategy also aimed at reducing the health gap between; low and high income families, professional and unskilled individuals and differing geographical areas of the nation.	0
Saving Lives: Our Healthier Nation (UK)	1999	Public health strategy including obesity	The key aims of this public health strategy were to promote healthier living and reduce health inequalities. The focus of the strategy was targeting the major public health issues of cancer, heart disease and stroke, accidents and mental illness.	0
Towards A Healthier Scotland: A White Paper on Health (Scotland)	1999	Public health strategy including obesity	This white paper focused on reducing health inequalities, improving children’s health and initiatives to drive down cancer and heart disease rates. Focus was placed on tackling unhealthy lifestyles of individuals such as poor diet and lack of exercise and alcohol and drug misuse.	“Alcohol misuse is linked with crime, lower achievement, poor mental and physical health.”
Choosing Health: Making healthier Choices Easier (UK)	2004	Public health strategy including obesity	This aim of this white paper was to set out how the UK Government would provide opportunities, support and information for individuals to make healthier choices. Obesity reduction formed one part of the Government’s priorities which also included; reducing the numbers of the people who smoked, increasing physical activity of the population, encouraging and supporting sensible drinking and improving mental health.	0
Choosing a Better Diet: A Food and Health Action plan (UK)	2005	Obesity strategy	This paper outlined how the UK Government was going to deliver on commitments to nutrition including restricting advertising and promotion of foods to children as well as obesity education and prevention. Other aims of this strategy included encouraging healthy eating behaviours in children and young people and the promotion of opportunities for healthy eating in the workplace.	“Alcoholic drinks, while not classed as a nutrient, can for many people make a significant contribution to total energy intake.” “As part of our work on obesity, however, we will consider how best to increase public awareness of the energy content of alcoholic drinks.”
Healthy Weight, Healthy Lives: A Cross- Government Strategy For England (UK)	2008	Obesity strategy	One of the key aims of this obesity strategy included focusing on childhood obesity. The UK Government introduced measures to increase fruit and vegetable consumption within schools as well as increasing the number of school children participating in sports. Other key aims of this obesity strategy included promoting healthier food choices and decreasing the hours of sedentary behaviour of the population.	“Alcohol consumption is also a part of an individual’s calorie intake, and so the rising trend in consumption also contributes to excess weight.”
Food Matters: Towards a Strategy for the 21st Century (UK)	2008	Food Policy	Improved diet was one aspect of this policy paper but consideration was also given to food production and the wider implications for the economy, society and the environment. The UK Government’s main objectives for food were to secure a further transition to healthier diets and a more environmentally sustainable food chain.	0
Healthy Eating, Active Living: An action plan to improve diet, increase physical activity and tackle obesity (2008–2011) (Scotland)	2008	Obesity strategy	This strategy aimed to reduce the prevalence of obesity in the Scottish population through working with industry to promote healthier food choices and creating environments that encouraged more active lifestyles.	0
Recipe For Success: Scotland’s national food and drink policy (Scotland)	2008	Food and drink policy	This paper outlined how the Scottish Government planned to support the growth of the food and drink industry as well as working with retailers on specific guidance on how to prepare low-cost healthy meals.	0
Healthy Lives, Healthy People: our strategy for public health in England (UK)	2010	Public health strategy including obesity.	This white paper introduced the UK Government’s new strategy to tackle public health issues within England which included the creation of the new public health service, Public Health England.	0
Preventing Overweight and Obesity in Scotland: A Route Map Towards Healthy Weight (Scotland)	2010	Obesity strategy	This strategy aimed to reduce obesity levels of the Scottish population by controlling exposure to, demand for and consumption of excessive quantities of high calorific foods as well as increasing opportunity for physical activity within daily life.	“Obesity occurs when energy intake from food and drink consumption, including alcohol, is greater than energy requirements of the body’s metabolism over a prolonged period, resulting in the accumulation of excess body fat.” “We need to reduce the energy intake of Scotland’s population. This means consuming smaller quantities of energy from food and drink, including alcohol.”
Healthy Lives, Healthy People: A Call to Action on Obesity in England (UK)	2011	Obesity strategy	This paper outlined the UK Government’s new approach for tackling obesity including empowering individuals to make healthier choices, allowing industry to play their part through the Responsibility Deal and building the evidence base on effective and cost effective actions to reduce obesity levels.	“Alongside the Food Network, the Alcohol Network of the Responsibility Deal is looking to develop further pledges which could also support calorie reduction. Calories from consumption of alcoholic drinks account for over 9% of all calorie intake for 16–64-year-olds who drink alcohol, and more for those who drink at higher levels. A measure of spirits has more calories than the equivalent amount of single cream (25 ml of spirits at 40% abv = 56 kcal compared with 47 kcal for 25 ml of single cream). Heineken has already pledged to reduce the strength of a major brand. This aims to remove 100m units of alcohol from our consumption each year – the equivalent of 5.6bn calories.”
2010 to 2015 government policy: obesity and healthy eating (UK)	2011	Obesity strategy	This policy document outlined the actions the UK Government were taking to help people make healthier choices such as improving labelling on food and drink as well as building on the previously mentioned Public Health Responsibility Deal and how businesses were key partners in helping consumers to make healthier choices.	0
Childhood obesity: a plan for action (UK)	2016	Childhood obesity strategy	This strategy outlined the UK Government’s plan for action to significantly reduce childhood obesity. This included the introduction of the Soft Drinks Industry Levy as well as committing to increasing the nutritional value of school meals.	0
Health Matters: Obesity and the Food Environment (UK)	2017	Obesity strategy	This obesity strategy focused on increasing physical activity levels of the population as well as improving consumers access to healthier food options in the out of home environment	0
Childhood Obesity: A Plan for Action, Chap. 2 (UK)	2018	Childhood obesity strategy	This strategy outlined the actions the UK Government planned to take to achieve its goal of halving childhood obesity and reducing the gap in obesity between children from the most and least deprived areas by 2030. This included reducing the sugar content in the food that children eat as well as reducing the marketing and promotion of unhealthy food and drink to children.	0
A healthier future: Scotland’s diet and healthy weight delivery plan (Scotland)	2018	Obesity strategy	This strategy focused on the public health issue of childhood obesity as well as improving the food environment to support healthier choices. This strategy also sought to decrease diet-related health inequalities.	0
Advancing Our Health: Prevention in the 2020s (UK)	2019	Public health strategy including obesity	The obesity aspect of this strategy continued to focus on restricting advertising of HFSS as well as improving accessibility and affordability of healthier foods.	0
Tackling Obesity: Government Strategy (UK)	2020	Obesity strategy	This UK Government strategy focused on providing consumers with easy access to information to make healthy choices. This included the proposal of the consultation on making companies provide calorie labelling on all pre-packaged alcohol they sell.	“Of course, it is not just food that adds to our energy intake, alcohol is highly calorific too. It has been estimated that for those that drink alcohol it accounts for nearly 10% of the calories they consume. We know that each year around 3.4 million adults consume an additional day’s worth of calories each week from alcohol, nearly an additional 2 months of food each year. Despite this, in the UK alcoholic drinks are not required to provide any information about how many calories they contain. We also know that the public is largely unaware of the calorific content of alcohol. Surveys have shown that up to 80% of adults did not know the calorie content of common drinks. Therefore, we will consult before the end of the year on our intention to make companies provide calorie labelling on all pre-packaged alcohol they sell, so when consumers shop for alcohol, they have all the information they need to make healthier choices. The consultation will also cover introducing calorie labelling on alcoholic drinks sold in the out-of-home sector, for example bought on draught or by the glass, as we have done with our measures on food and non-alcoholic drink outlined above.”

These policy documents were initially read and searched for any references to alcohol as an obesity policy issue and relevant quotations relating to these were extracted and tabulated. A five-stage reflexive thematic analysis, as outlined by Braun and Clarke [25], was undertaken to analyse key foci and themes within these alcohol policy documents using Nvivo11 qualitative data analysis software.

A stakeholder analysis was undertaken to identify public health actors and commercial organisations engaged in alcohol and obesity policy debates [26] by searching the respondent lists of obesity-related UK and Scottish Government consultations. The included stakeholders are outlined in Table 2. Obesity consultation responses by these organisations were accessed on the UK and Scottish Government websites, or on the organisations’ own sites. This allowed a cross table (Table 3) to be created to visualise which stakeholder had responded to each government obesity consultation.

**Table 2:** Key alcohol and obesity policy stakeholders

Alcohol companies, trade associations & social aspects organisations	Public health actors
AB InBev	Action on Sugar (AOS)
British Beer and Pub Association (BBPA)	Alcohol Focus Scotland (AFS)
Diageo	Alcohol Health Alliance (AHA)
Drinkaware	British Heart Foundation (BHF)
Heineken UK	Cancer Research UK
National Association of Cider Makers (NACM)	Diabetes Scotland
The Portman Group (PG)	Obesity Action Scotland (OAS)
The Wine and Spirit Trade Association (WSTA)	Obesity Health Alliance (OHA)
The Scotch Whisky Association (SWA)	Royal Society of Public Health (RSPH)
	Scottish Health Action on Alcohol Problems (SHAAP)
	Slimming World

Identified stakeholders’ websites were searched for materials such as press releases, on the topic of alcohol and obesity. In some cases, the “Wayback Machine” internet archive was utilised to gain access to items that had been taken off the current version of the websites. These consultation responses and press releases were analysed qualitatively along with government policy documents and strategies using the same method of qualitative thematic analysis described above.

## Results

The results of this study are presented in three sections. The first examines the engagement of UK and Scottish Government obesity policy documents with alcohol. The second and third sections focus on the respective policy positions of public health NGOs and alcohol industry actors on this topic.

### Obesity policy engagement with alcohol

The 19 UK and Scottish Government documents that were included in this analysis were a combination of obesity specific strategies, food and drink policies and general public health strategies. Engagement with alcohol in obesity policy documents was limited in both Scotland and England, however it was not completely absent. Alcohol was first identified as a policy problem in England’s 1999 public health strategy [27]. However, it was not until the 2005 obesity strategy [28] that the energy content of alcohol was explicitly linked to weight gain, although this was not accompanied by specific policy measures to combat this issue. A similar approach was evident in the 2008 [29] and 2010 obesity strategies [30].

Alcohol was not recognised in Scotland’s obesity policy strategies until the 2010 obesity route map [31]. However, the Scottish Government’s alcohol strategy [32], published two years previously, had highlighted that a reduction in alcohol consumption would be beneficial in the fight against obesity. Scotland’s 2018 obesity reduction strategy [13] linked plans to improve diet and overweight with the Scottish Government’s five other public health strategies including alcohol prevention, suggesting an integrated approach to obesity. However, it contained no specific measures to address alcohol as an obesity risk factor, for example raising public awareness of the calorie content of alcohol via calorie labelling on alcoholic beverages.

The UK Government’s 2020 strategy, which sought to tackle obesity by providing consumers with easy access to information to make healthy choices [14], recognised the “highly calorific” nature of alcoholic beverages and low levels of public awareness about this, proposing a consultation on alcohol calorie labelling. However, at the time of writing, it is yet to be actioned. In Scotland, a similar consultation for calorie labelling on alcoholic drinks was proposed in 2022 [33] but, as in England, is yet to be released. Table 1 (above) presents all references to alcohol in relation to obesity within the policy documents examined.

### Industry partnership

In the policy documents examined, industry actors were consistently recognised as a key partner to both the UK and Scottish Governments in their efforts to improve population health. In England’s 2004 public health strategy, which included measures such as labelling foods to indicate fat, salt and sugar content [34], the CEO of the alcohol industry-funded Portman Group was quoted affirming their “strong shared agenda with government in promoting responsible drinking” and that they were “fully committed to working with government and the public health community towards our common objective.” The explicit reference to this organisation, which is predominantly funded by the alcohol industry, in a governmental strategy highlights the significant level of partnership between industry actors and government [19].

Similarly, Scotland’s 2008 food and drink policy [42] set out how the government would support the growth of the food and drink industry, while educating people on making healthy and sustainable food choices. Furthermore, it stated that they would “work with the drinks industry to take forward collaborative work around the responsible drinking agenda” via the Scottish Government Alcohol Industry Partnership; an established forum which formalised alcohol industry engagement with policymaking [23].

In England, the *Public Health Responsibility Deal* (PHRD) involved the UK Government working in collaboration with business and public health actors to develop voluntary, self-regulatory measures to tackle obesity and excessive alcohol consumption including product labelling [30]. Heineken, for example, pledged to reduce the strength of a major brand which aimed to remove 100 million units of alcohol from the population’s consumption each year which is equivalent to 5.6 billion calories. However, evidence indicated that the industry failed to achieve this target [35]. England’s 2011 obesity strategy [36] made specific reference to the work of the Alcohol Network of the PHRD, which looked to develop voluntary targets that could support calorie reduction. England’s 2020 obesity strategy [14] showed the first signs of divergence from the voluntary and industry-partnership approaches taken the UK Government with the proposal for a consultation on mandatory calorie labelling on all pre-packaged alcohol.

### Childhood obesity

A key focus of both the UK and Scottish Governments’ obesity strategies was reducing the prevalence of childhood obesity. In the foreword of Scotland’s 2018 obesity strategy [13], then First Minister^2^, Nicola Sturgeon, pledged to halve childhood obesity by 2030, placing this at the heart of the delivery plan. The UK Government also focused on the reduction of childhood obesity, publishing two strategies explicitly on this in 2016 [37] and 2018 [38].The first of these included plans for the implementation of the Soft Drinks Industry Levy (SDIL) [37]. They made the same pledge as the Scottish Government to halve childhood obesity rates by 2030, citing again its lifelong impacts. To do so, they proposed banning the sale of energy drinks to children, due to their high sugar content, and improving nutrition standards in schools.

### Public health NGOs

Several public health actors sought to highlight that alcohol is highly calorific, can lead to weight gain, and thus should not be exempted from relevant obesity policy measures. They voiced their opinions in several ways such as reports and consultation responses. Table 3 outlines obesity related consultations in Scotland and England and indicates which stakeholders responded to the consultation.

**Table 3:**
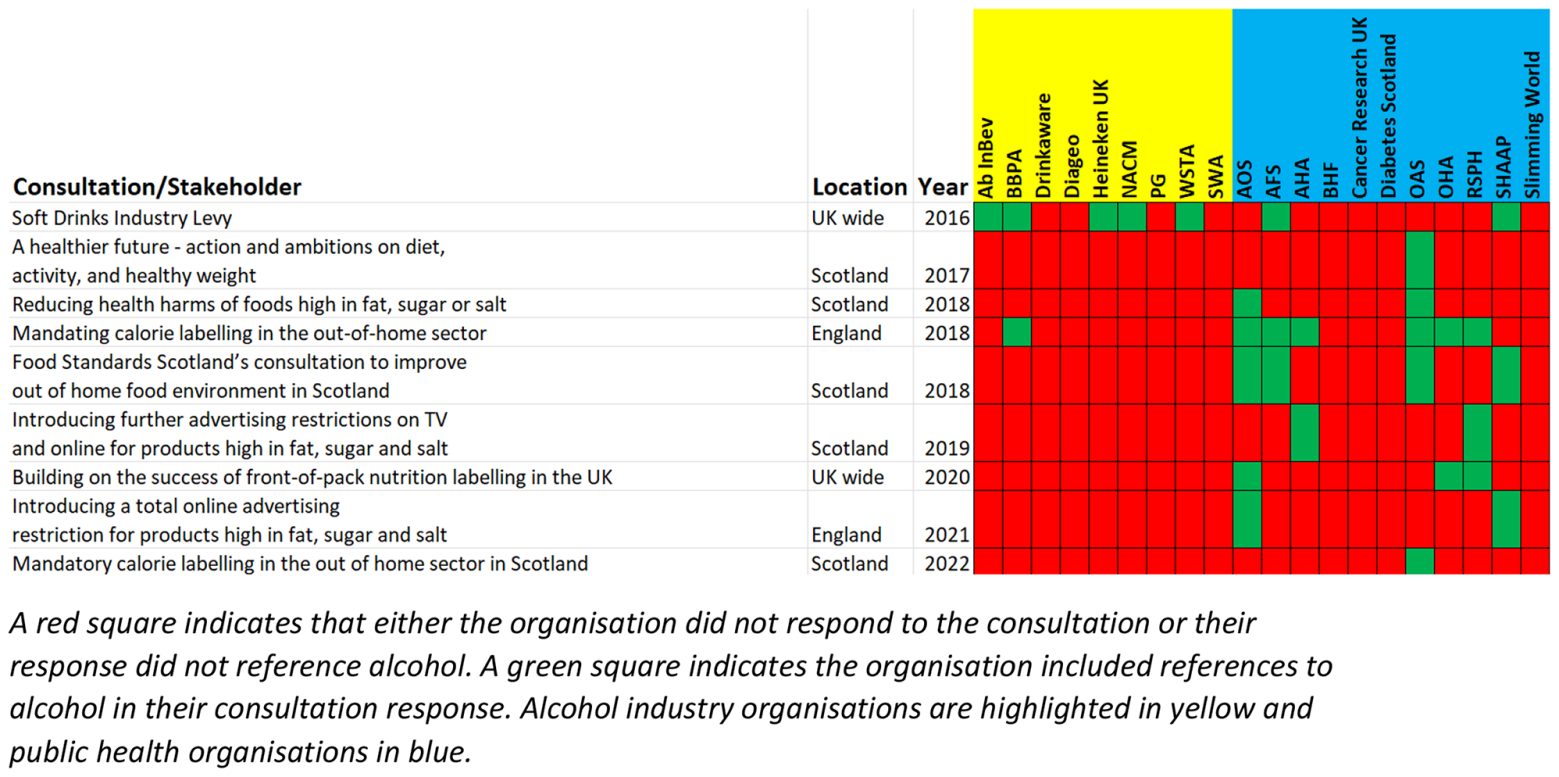
Stakeholder responses to UK and Scottish Government obesity-related consultations

In their response to the Scottish Government’s 2019 consultation [39] Scottish Health Action on Alcohol Problems (SHAAP) expressed their concern that there was no reference to alcohol in proposals to restrict television and online advertising of products high in fat, sugar and salt (HFSS) [40], arguing that alcohol is “toxic, calorific and addictive.” The Alcohol Health Alliance also strongly disagreed with the omission due to the “links between alcohol and obesity” [41]. SHAAP repeated their concern about the exemption of alcohol from further proposed advertising restricting obesity legislation in their response to the UK government’s consultation [42] in 2021, highlighting the anomaly that while proposals were targeting advertisements for HFSS products “alcohol, a substantial contributor to calorie intake, is excluded.”

SHAAP also called on the UK and Scottish Governments to introduce improved nutritional information on alcohol labelling as this would allow individuals to make better informed choices [43]. A January 2020 report by Action on Sugar [44] highlighted the fact that sugar-sweetened alcoholic drinks had been completely omitted from obesity policy, including nutritional labelling, arguing that:

it is vital that customers be afforded an informed choice when purchasing food or drinks – there is no good reason why this should be any different simply because these drinks contain alcohol.

In May 2021 Alcohol Health Alliance wrote a letter to then Secretary of State for Health, Matt Hancock to express their “support for the inclusion of calorie and health information on alcohol products” [45]. The letter argued that existing labelling requirements for alcoholic beverages failed to provide consumers with the necessary information to make informed decisions about what they drink. The letter was co-signed by other public health bodies including The Royal Society for Public Health, Cancer Research UK and Alcohol Focus Scotland (AFS). The proposal echoed previous calls for alcohol labelling in a 2017 report by the Royal Society of Public Health [46].

Public health NGOs also highlighted the lack of public awareness of the calorie content of alcohol in their consultation responses. In their response to *Restricting Alcohol and Advertising and Promotion* consultation in 2022 [47, 48], Obesity Action Scotland cited evidence that only a quarter of people could correctly identify the number of calories in a medium glass of wine, a pint of beer and a standard measure of spirit. In addition, they highlighted the high levels of public support for this policy to be implemented [48]. In May 2021, AFS revealed the results of a YouGov poll that found 59% of people wanted calorie information to be included on alcohol labels, while 48% supported the inclusion of sugar content [49].

Cancer Research UK articulated support for alcohol calorie labelling by signing the joint letter to Health Secretary, Matt Hancock, cited above. However, on their website, and in their soft drinks industry levy consultation response, they did not explicitly engage with the link between alcohol and obesity even though both are prominent risk factors for cancer [50, 51]. There was no further evidence identified of Cancer Research UK supporting the inclusion of alcohol into obesity policy in the documents analysed here, despite its potential to decrease population-level obesity, which they identify as a key the risk factor for many cancers [52]. The British Heart Foundation recognised the link between alcohol consumption and weight gain on their website [53], but there was no evidence found in the analysis of this dataset of them lobbying to raise awareness of energy-dense alcoholic drinks as a risk factor of obesity, or for the introduction of relevant policies to address consumption.

### A whole systems approach

Public health NGOs advocated for a “whole systems” approach linking the issues of excessive alcohol consumption and obesity. SHAAP expressed their views that the energy content of alcohol should be considered as part of the solution to the obesity epidemic [43]. In response to *Food Standards Scotland’s* 2018 consultation on improving out of home food environment [43, 54] they argued that two thirds of Scotland’s population was overweight and obese, and that “radical measures” were required to improve the dietary information consumers received in order to address this. They noted the importance of alcoholic drinks and their energy content as “an important part of this mix” [43].

In 2023 in a blog post Diabetes Scotland supported AFS’s aim to reduce alcohol-related harm arguing that “type 2 diabetes, alcohol problems and obesity are all part of a wider public health challenge which can’t be tackled in isolation” urging the incoming First Minister, Humza Yousaf, to “take every opportunity in his new role to support whole system approaches to these issues” [55].

### The ineffectiveness of self-regulation

While policy documents placed a strong emphasis on industry partnership, public health actors did not believe that self-regulation was effective, and that government intervention was, therefore, required. It is important to note here that self-regulation specifically refers to the calorie labelling on alcoholic beverages as opposed to general health alcohol warning labels. In their 2019 consultation response to Food Standards Scotland proposal to improve the out of home food environment [54], SHAAP were critical of self-regulation stating that voluntary regulation by members of the alcohol industry in UK had been “proven not to work,” citing evidence that only 1% of alcohol product labels reviewed provided calorie information [43].

In 2020 the Alcohol Health Alliance released the report *Drinking in the Dark*, which outlined how alcohol labelling was failing consumers [56]. They argued that alcohol manufactures had “failed to tackle the inconsistency, inadequacy and poor quality of alcohol labelling” [56]. They found that 56% of labels analysed included no nutritional information, 37% of labels listed the calories without further information and only 7% displayed full nutritional information including calories. This, they argued, demonstrated the consistent failure of voluntary approaches over the preceding 20 years.

### Childhood obesity

Public health NGOs consistently argued that the UK and Scottish Government strategies on reducing childhood obesity should not exclude alcohol. While recognising that the aim of this strategy was focussed at minors unable to purchase alcohol legally, SHAAP argued that they may nevertheless access and consume these products. Consequently, they argued in their response to the UK Government’s consultation on HFSS food advertising [39] in 2019 that “to ignore/ exempt alcoholic drinks with a high calorie and sugar content to which [children] may be exposed makes no sense.” Similarly, when the consultation on the SDIL was released in 2016 and sought respondents’ thoughts on the inclusion of low and no alcohol beers [57]. AFS called for alcoholic drinks to be included due to evidence of under-18s consuming alcoholic beverages, especially highly calorific and sugary ciders [56].

In a 2020 blog post, Action on Sugar [58] argued that while “it may be easy to assume that alcohol is not a major contributor to child obesity, […] in the UK almost half of 15 year olds thought it was acceptable to drink,” and that teenagers in Scotland were more likely to consume alcohol than smoke cigarettes. Similarly, in their June 2021 consultation response to the UK Government consultation on total restriction of online advertising for products high in fat, sugar and salt 2021 [59], they cited evidence that highly sugary, brightly coloured ready to drink products were particularly appealing to adolescents [60].

### Alcohol industry actors

Throughout the documents analysed, alcohol industry actors in both England and Scotland argued against the identification of alcohol consumption as an obesity policy issue. Several alcohol companies called for the exclusion of low and no alcoholic beverages from the SDIL and opposed mandatory calorie labelling for alcoholic beverages. In addition, they sought to position themselves as part of the solution to the obesity epidemic, arguing that voluntary regimes for calorie labelling and product reformulation were the correct policy responses.

### Alcohol industry lobbying

Alcohol industry actors argued that low/no alcoholic drinks should not be included in the SDIL due to the focus of the policy on childhood obesity. While products with less than 0.5% ABV are not technically classified as alcoholic products, there is widespread application of age restrictions on the sale of products by on- and off-trade retailers. Similarly, producers claim that they are marketed exclusively at adult consumers as an alternative product. As Heineken UK argued:

These products are about helping adults who want to moderate their alcohol consumption. They are not targeted at under 18s and therefore should be exempt from the levy [61].

Similarly, industry representatives, including The British Beer and Pub Association and The Wine and Spirits Trade Association, called for low and no alcoholic drinks to be exempted from the SDIL, arguing that to include them would be a “major blow” to the ability of this emerging product category to deliver “positive public health benefits” [62]. They cited the PHRD and drinks industry pledges to reduce total alcohol units sold in the UK, in part by increasing the level of low and alcohol-free alternatives available, as a rationale for excluding these products from the SDIL [62]. In increasing the cost of low and no alcohol products, they argued, the levy could disincentivise their consumption in place of higher-alcohol products [62].

### Calorie labelling

Industry actors used voluntary labelling commitments to argue against the inclusion of alcoholic drinks in mandatory labelling regimes. For example, in their response to a 2013 consultation on front of pack nutrition labelling [63], the British Beer and Pub Association acknowledged that consumers are “generally unaware of the actual nutritional content of beer and as such do not have an informed view of the calorie content” [64], and they declared that they would be “prepared to work with UK Government in their approach to provide accurate and relevant front of pack information on a voluntary basis to UK consumers” [64]. This indicated their belief that alcoholic products should be exempt from government regulation and their preference for a partnership-based approach.

In March 2018 the Scotch Whisky Association illustrated their commitment to voluntary calorie labelling by announcing that whisky bottles would feature calorie information by 2022 [65]. In their 2021 market review the Portman Group cited evidence from their own study that found 47% of alcoholic drinks had calorie labelling on, illustrating that “voluntary efforts concerning calorie labelling are bearing fruit” [66]. Furthermore, their CEO called for the removal of mandatory labelling from the proposed UK Government consultation addressing alcohol labelling within the overall obesity strategy [14], again citing the above evidence of effective calorie labelling. They believed that this “is a significant achievement that the industry is delivering on its commitment to ensuring high standards of voluntary best practice” [66].

Similarly, several of the alcohol industry actors, such as the Wine and Spirits Trade Association, included calorie counting tools within their “responsible drinking” sections of their websites [67]. Allowing consumers to see the calorie content of a variety of alcohols online, it could be argued, was a way of obviating the need for on products calories labelling, or a basis for arguing that this was the case. Similarly, while the industry-funded Drinkaware website recognised the potential role of alcohol consumption as a risk factor for obesity and encouraging consumers to reduce calorie intake by reducing their alcohol consumptions, they did not advocate for mandatory calorie labelling for alcoholic beverages [68, 69]. From the analysis of members of the alcohol industry it was clear that they were in strong opposition to mandatory calorie labelling for alcoholic beverages.

### Alcohol’s benefits to society

Alcohol was highlighted by members of the alcohol industry as being beneficial to society which could be a potential reason why the UK and Scottish governments have not intervened with population-wide measures such as calorie labelling as they don’t view alcohol as an exclusively harmful product. The British Beer and Pub Association responded to leaked proposals for calorie labelling on pints in 2021 by saying that “the pub has an important role to play in tackling loneliness and improving mental health” [70].

Similarly, in 2020 the Portman Group issued a press release [71] that said that there “are clear mental health benefits from spending time with friends and many people are likely to take advantage of the chance to socialise over a pint or a glass of wine.” Additionally, on the “responsible drinking” section of the Scotch Whisky Society website it is reported that whisky can “play a positive role in social occasions and celebrations” indicating that alcohol is key part of social occasions, deflecting attention from the negatives of this product. By portraying this positive picture of alcohol and all the benefits it can bring to society such as improving mental health and being a necessity for celebrations, the alcohol industry is shifting focus away from the harm this product can cause, including weight gain caused by the high calorie content.

## Discussion

Approaches to the inclusion of alcohol in the obesity policy debate were similar in both England and Scotland. This was somewhat surprising given the significant divergence of Scottish and English alcohol policy exemplified by the adoption of minimum unit pricing in the former and the PHRD in the latter [72]. This lack of policy divergence could be due to the “chilling effects” arising from the costly legal battles with members of the alcohol industry over the implementation of minimum unit pricing, and the consequent reluctance of the Scottish Government to adopt new alcohol policy measures [21]. However, it may also be the consequence of a siloed approach to health policy and the path dependency of reductionist, yet deeply embedded, understandings of obesity, which continue to constrain the policy debates in both contexts. Before 2020, alcohol remained unacknowledged as a risk factor in UK and Scottish obesity policy documents, and no strategies were implemented to raise the public awareness of the calorie content of alcoholic beverages. In more recent policy documents in both England and Scotland, alcohol does feature as an obesity policy problem, but engagement remains limited and alcohol-specific policy interventions appear to be low priorities. For example, proposed consultation on calorie labelling for alcoholic beverage have been mooted by both the Scottish and UK Governments but, at the time of writing, these are yet to be brought forwards in either context.

Both the Scottish and UK Governments placed a particular emphasis on childhood obesity and introduced several strategies aimed specifically at improving children’s health such as the ban on advertising of HFSS food and drink in children’s media in 2017 and ending the sale of energy drinks to under-16s in 2020. In addition, other policies with wider population level effects were also framed as anti-child-obesity measures. This perhaps reflects the wider acceptability of interventions to protect children and could thus serve to increase political support for novel, and potentially controversial, policies such as the SDIL. However, despite pushback against this by public health NGOs, this framing created the pretext for industry actors to oppose the inclusion of both alcoholic and low and no alcohol alternatives – which, while not officially classified as alcohol products, are largely unavailable for sale to under 18s – within the policy.

In keeping with the rapidly expanding literature on the commercial determinants of health, and corporate political strategies of the alcohol [73] and other industries [74], industry actors invested substantial effort into portraying themselves as key partners to government and in advocating for their favoured policy solutions. They used voluntary calorie labelling to argue against the introduction of mandatory labelling regimes by UK and Scottish Governments. In keeping with previous studies [73, 75, 76], they deflect attention away from the product, as well as highlight the beneficial role it plays on society, and instead place responsibility for harms on consumers, arguing that policies should target the minority of drinkers who they claim abuse alcohol. Members of the alcohol industry also showed their influence over obesity policy-making through the successful lobbying of the UK Government to remove low and no alcohol products from the SDIL. It is possible to detect a shift in UK obesity policy towards more interventionist policies (e.g. the SDIL) after the disbanding of the PHRD. This suggests that the government had begun to identify that previous collaborative partnerships were unable to deliver the desired improvements in public health, and that mandatory regulation may be required. Yet the extent of this change should not be overstated, and the focus of obesity policy remains principally aligned with industry-favoured policy prescriptions. Similarly, the alcohol industry continues to be seen as a key stakeholder in the formulation of health policy in Scotland and England.

Some public health NGOs, by contrast, have advocated for the inclusion of alcohol in relevant obesity policies from 2016 as part of a systems-based approach to tackling non-communicable disease risk factors. They argued that the lack of public awareness of the calorie content of alcoholic beverages, due in part to the ineffectiveness of the current, voluntary alcohol labelling regime, required government intervention and mandatory labelling. Several Public health NGOs also challenged arguments about the exclusion of alcohol products (including low and no alcohol alternatives) from childhood anti-obesity measures, arguing that such exemptions were unjustifiable since children, despite age restrictions, consume alcohol and, when they do so, are particularly likely to choose calorific, high-sugar ciders and ready-to-drink beverages [58]. It is noteworthy that an alcohol harm charity such as SHAAP was engaging so vocally on obesity policy issues, believing that the issues of excessive alcohol consumption and obesity should be tackled in unison. The joint approach to lobbying the Scottish Government adopted by AFS and Diabetes Scotland indicates that the public health NGOs are acutely aware of the potential impact that alcohol can have on weight gain. They believe that a “two birds, one stone” approach should be taken by treating the public health issues of obesity and alcohol problems simultaneously. This type of joined up thinking offers guidance for both health advocates and policy-makers seeking effective policy responses to improve population health.

### Strengths and limitations

An extensive array of literature has investigated how members of the alcohol industry engage with alcohol policy [73], but less attention has been placed on how they seek to engage with and shape obesity policy. This article addresses that gap and examines the position of public health NGOs, alcohol manufacturers, alcohol industry representatives and social aspect organisations on this topic. Through the inclusion of obesity policy documents from 1999 onwards, comparisons could be drawn between England and Scotland as this was the year that Scotland gained autonomy over decisions regarding health and social care including obesity policy. The study draws on a large data set including obesity policy documents, consultation responses and press releases, which allowed for a detailed description of how each stakeholder engaged with the issue of alcohol in the obesity policy setting.

While the study finds considerable alignment between obesity policies and the preferred approaches of the alcohol industry, it is unable to identify specific evidence of industry influence over policymaking based on the secondary data analysed here. To investigate policy-making process would require other methods. Interviews with relevant policy actors, for example, would have allowed the authors to examine attempts by different actors to influence policy processes, and their relative success. This analysis should form the basis of future studies.

## Conclusions

Having been largely absent until recent years, alcohol remains only a peripheral issue in UK and Scottish government obesity policies and obesity-relevant health strategies. There is an over emphasis on voluntary and partnership-based approaches with industry actors to the exclusion of more effective mandatory responses. The exclusion of alcoholic beverage from relevant tax and labelling regimes seems anomalous given the potential impact of alcohol drinks on calorie consumption and obesity. Public health NGOs clearly recognised the calorie content of alcohol and the impact that it can have on the obesity levels of the population. They have consistently argued that alcoholic drinks should not remain exempt from obesity policies, especially those targeting children as this demographic, although not legally, are still consuming alcoholic beverages. They argued that the deficit in awareness of the calorie content of alcoholic beverages from the public is due to the current ineffective, voluntary labelling regimes in England and Scotland. They firmly believe that a whole systems approach should be taken to the issues of excessive alcohol consumption and obesity, and repeatedly advocated for mandatory calorie labelling onto alcohol beverages.

On the other hand, alcohol industry actors continue to portray themselves as part of the policy solution to the obesity epidemic through voluntary calorie labelling and low-calorie alternative products. With the upcoming consultations on mandatory calorie labelling in both England and Scotland, it will be interesting to see if members of the alcohol industry continue to argue that voluntary labelling is effective, and that regulation is thus not required. Similarly, will the UK and Scottish Governments still consider them a key part of the policy solution, despite the limited success of voluntary approaches? Further research is needed to address these questions and to understand the influence of alcohol industry actors and public health bodies on alcohol as an obesity policy issue.

## Data Availability

Data analysed in this study are in the public domain and can be accessed via in text references.

## Footnotes

1 For clarification it is important to note that the UK Government sets policies for England, therefore throughout this text, when considering English obesity policy, the UK Government will be referred to as the relevant legislative authority.

2 The First Minister of Scotland is the head of the Scottish Government, chairing the Scottish Cabinet and responsible for the formulation, development and presentation of Scottish Government policy.

## Abbreviations

AFS: Alcohol Focus Scotland
HFSS: High in Fat, Sugar and Salt
PHRD: Public Health Responsibility Deal
SDIL: Soft Drinks Industry Levy
SHAAP: Scottish Health Action on Alcohol Problems
